# Early gut microbiota transplantation in broiler chicks exerts effects on intestinal health depending on the donor’s microbial composition

**DOI:** 10.1016/j.psj.2026.106999

**Published:** 2026-04-22

**Authors:** Haoran Zhao, Muhammad Zeeshan Akram, Luke Comer, Matthias Corion, Elena Fako, Nadia Everaert

**Affiliations:** Nutrition and Animal-Microbiota Ecosystems Laboratory, Department of Biosystems, KU Leuven, Heverlee, Belgium

**Keywords:** Broiler, Fecal microbiota transplantation, Gene expression, Tryptophan

## Abstract

Fecal microbiota transplantation (**FMT**) is a promising strategy for modulating the gut microbiota to enhance broilers’ health. This study investigated the effects of FMT of broilers (FMT1), laying hens (FMT2), and broiler breeders (FMT3) as donor on the intestinal health of chicks as recipients. A total of 144 newly hatched Ross 308 male broilers were randomly assigned to four groups and reared until D 14. For the first three days after hatching, the control group received 1 mL of saline daily via oral gavage, while the FMT groups received their respective inocula. Body weight, intestinal length, and ileal histomorphology were assessed on D 5, D 7, and D 14. In addition, on D 7 and D 14, ileal tissues and serum were collected. The expression of critical genes related to barrier function, nutrient transport, mitochondrial function, antioxidant defense, signal transduction, innate immunity, cytokine response, and programmed cell death were measured using high-throughput qPCR. Furthermore, serum metabolites related to tryptophan metabolism were also determined. Group differences were analyzed using one-way ANOVA with Tukey’s post-hoc test. Partial Least Squares Regression (**PLSR**) model was used to evaluate the interaction between observed changes and dominant microbiota. No significant effects on body weight or intestinal length were observed. On D 7, FMT1 induced innate immune stress, characterized by upregulated Toll-like receptor signaling, apoptosis, and mitochondrial biogenesis. In contrast, FMT2 and FMT3 improved intestinal histomorphology. Notably, FMT2 enhanced antioxidant defenses and changed tryptophan metabolism, while reducing reliance on glucose transporters. Meanwhile, FMT3 suppressed pyroptosis and enhanced barrier integrity. PLSR analysis identified 9 features that contribute over 30% of observed variations, with *Olsenella, Blautia*, and unclassified *Lachnospiraceae* being the most pronounced. By D 14, most effects had diminished, although antioxidant effects in FMT1 and immune markers in FMT2 and FMT3 persisted. Overall, FMT exerted donor-dependent patterns of influence on intestinal health, and our study proved programming of gut physiology through fecal microbiota transplantation.

## Introduction

The early post-hatch stage is the critical period for establishing a diverse gut microbiota in broilers, as microbial load is still low and intestinal ecological niches are not fully established (Dai et al., 2022; Zhao et al., 2025b). During this window, pioneer microbes colonize the gut and shape its initial microbial framework by competing for nutrients, secreting antibacterial substances, or modulating the host immune system (Zhao et al., 2025b). However, ubiquitous environmental pathogens such as *Escherichia coli* can disrupt this delicate colonization process, posing a challenge to the maturation and stable development of the intestinal microbiota in broiler chickens (Kers et al., 2018). The gut microbiota plays diverse and critical roles by synthesizing essential vitamins, metabolizing dietary fibers into short-chain fatty acids (**SCFA**) that serve as key energy substrates for intestinal epithelial cells, and modulating immune responses to reinforce the mucosal barrier, thereby contributing significantly to host health and homeostasis (Zhao et al., 2025b). Studies further show that its early composition substantially shapes the host’s microbial structure and physiological state during later growth stages (Dai et al., 2022; Gensollen et al., 2016). Therefore, the early post-hatch window offers a crucial opportunity to modulate the gut microbiota and promote an optimal microbial structure for healthy growth in broilers (Gensollen et al., 2016; Zhao et al., 2025b).

Fecal microbiota transplantation (**FMT**) aims to transfer healthy microbial communities to recipients to promote optimal physiological functions (Zhao et al., 2025b). Recent studies have increasingly explored FMT applications in poultry, with most demonstrating that it effectively alters the gut microbial composition of recipient chickens (Elokil et al., 2022; Yan et al., 2021; Yu et al., 2021; Zhang et al., 2022a, 2022b). Several studies suggest that FMT can enhance broiler growth performance, including body weight and feed efficiency (Chen et al., 2024; Elokil et al., 2022; Fu et al., 2022; Khalid et al., 2024; Ma et al., 2023; Siegerstetter et al., 2018; Yu et al., 2021; Zhang et al., 2022b), likely by introducing beneficial gut microbiota that subsequently produce metabolites, such as SCFA, supporting intestinal health (Zhao et al., 2025b). Given the critical role of microbiota in the development of the host’s intestinal immune system, FMT has also been used to improve gut immune function in chickens (Ma et al., 2023; Zhang et al., 2022a). However, despite these positive effects, some studies indicate that FMT may fail to modify the recipient’s phenotype or even exert negative impacts on gut health, such as decreasing body weight, disrupting the intestinal barrier, and inducing intestinal inflammation (Chen et al., 2025a, 2025b). These contradictory findings highlight the considerable variability in FMT efficacy.

The effects of FMT on chicken gut health have been reported, including improvements in intestinal histomorphology (Yan et al., 2021; Zhang et al., 2022a), increased tight junction expression (Zhao et al., 2025b), and enhanced innate immune responses (Song et al., 2023; Zhang et al., 2022a; Zhou et al., 2021). However, available studies remain limited, and findings are inconsistent (Zhao et al., 2025b). The gut microbiota also plays a critical role in regulating mitochondrial function in intestinal cells (Litvak et al., 2018; Zhang et al., 2022c), but evidence on how FMT influences mitochondrial activity in chickens is scarce. In addition, growing evidence suggests that the gut microbiota actively participates in tryptophan metabolism by directly converting tryptophan into indole derivatives (Gao et al., 2020; Huang et al., 2024). Furthermore, it can influence host metabolic pathways, including the serotonin and kynurenine pathways, by modulating key host enzymes such as indoleamine 2,3-dioxygenase and tryptophan hydroxylase (Agus et al., 2018; Gao et al., 2020). These processes have been shown to influence immune responses, antioxidant capacity, and epithelial barrier function, thereby affecting intestinal homeostasis (Agus et al., 2018). Nevertheless, the role of FMT in shaping tryptophan metabolism has received little attention. Overall, the mechanisms of FMT on gut health in chickens remain poorly understood (Zhao et al., 2025b). Importantly, these effects are likely influenced by the microbial composition of the donor.

Despite growing evidence that FMT can influence various gut functions in chickens, it remains unclear whether donor-specific microbial composition drives these effects. Therefore, we hypothesized that the specific microbial composition of the FMT donor drives distinct effects on recipient gut health. To test this hypothesis, this study comprehensively elucidates the effects of FMT from different donor sources on gut function in chickens from the perspectives of ileal histomorphology, barrier function, nutrient transport, mitochondrial function and antioxidant defense, signaling pathways and innate immunity, programmed cell death, and tryptophan metabolism, providing a theoretical foundation for further optimizing FMT applications in poultry.

## Materials and methods

### Ethical approval

The animal experiment conducted in this study received approval from the Katholieke Universiteit Leuven’s Animal Ethics Committee (Protocol P134/2023, Belgium) and were performed within the animal facility of the Department of Biosystems at KU Leuven, Belgium.

### Donor selection

Fecal samples were obtained from three distinct donors with no prior antibiotic exposure: Ross 308 broilers (Broiler, aged 36 days); ISA Brown layers (laying hen, aged 30 weeks); and Ross 308 broiler breeders (Ross 308 broiler breeder, aged 35 weeks). Fresh fecal samples were collected from ten birds per donor type. The white, uric acid-rich portion of the droppings was discarded to minimize potential metabolic interference in recipient animals (Zhao et al., 2025b). Following transportation on ice, the collected feces were weighed in sterile tubes and then diluted in phosphate-buffered saline (**PBS**, pH 7.4) at a 1:6 (w/v) ratio. Then, the suspension was homogenized by blender (Mixwel, Alliance Bio Expertise), and centrifuged (800 × g, 10 min). The resulting supernatant was then filtered through a sterile 200 µm sieve (Retsch, Germany) to remove large particulate matter, producing a homogeneous inoculum. This final preparation was mixed with 10% sterile glycerol and stored at −80 °C for future use. The distinct microbial compositions of the feces from these three donor sources have been characterized via 16S rRNA sequencing in our previous work (Zhao et al., 2025a).

### Animals and housing

A total of 144 newly hatched male Ross 308 chicks were obtained from Belgabroed N.V. hatchery (Merksplas, Belgium). They were distributed into four experimental groups, each containing 36 birds. All chicks had been vaccinated *in ovo* against Newcastle disease and infectious bursal disease (Gumboro) on D 18 of incubation. For the first three days after hatching, the control group (**CON**) was given 1 mL of saline daily via oral gavage at 10:00 AM, whereas the FMT groups received their respective inocula at the same time, with the first gavage administered before the chicks had access to feed and water. The FMT groups were designated as FMT1 (receiving inoculum from Ross 308 broilers), FMT2 (receiving inoculum from ISA Brown laying hens), and FMT3 (receiving inoculum from Ross 308 broiler breeders). Chicks were initially housed under a temperature of 34 °C, decreased by 1 °C every 48 h. Lighting schedule was set to 23L:1D for the first 7 days, followed by an 18L:6D cycle for the remainder of the study. All chicks had ad libitum access to feed (Zhao et al., 2025a) and water and were reared until D 14.

### Sample collection

The first two weeks post-hatch were chosen as the experimental window because they represent the most dynamic and critical period for intestinal maturation and microbial succession in broilers. On D 5, D 7, and D 14, ten birds from each group, selected based on average body weight, were weighed and euthanized for sampling. Following electronarcosis, duodenum, jejunum, and ileum were separated, and their lengths were measured to track continuous growth trajectories. Because D 7 and D 14 provide a stable condition suitable for mechanistic evaluations, further serum processing and tissue sampling were restricted to these timepoints. Specifically for D 7 and D 14, blood was collected from the jugular vein, and serum was obtained by centrifugation (2000 × g, 15 min, 4 °C) and promptly stored at −20 °C until analysis. A 3-cm-long segment of the mid-ileum was carefully cut, emptied, gently washed with iced PBS, and transferred to a 4% formalin solution for tissue staining. The remaining ileal segment was then collected, snap-frozen, and stored at −80 °C for further analysis.

### Ileal histomorphology

The histomorphology analysis of ileal segments followed the methodology described in previous studies (Zhao et al., 2025a). Specifically, the ileal segments were dehydrated, cleared, and embedded in paraffin. Sections were then cut, deparaffinized in xylene, rehydrated, and stained with Alcian Blue-Periodic Acid Schiff. The stained sections were examined under a microscope at 20 × magnification. Villus height (**VH**) and crypt depth (**CD**) were measured in ten well-oriented villi per section using NDP.view2 software (Hamamatsu Photonics K.K., Hamamatsu, Japan). The villus height to crypt depth (**V/C**) ratio was also calculated.

### RNA extraction

Total RNA was extracted from ileal tissues using the ReliaPrep™ RNA Miniprep System (Promega, Madison, WI) following the manufacturer’s protocol. RNA concentration and quality were measured with a NanoDrop 2000 spectrophotometer (Thermo Fisher Scientific, Waltham, MA), and integrity was confirmed by 1% agarose gel electrophoresis.

### Primer design and validation

This research focused on analyzing 89 genes associated with diverse ileal physiological processes (Supplementary file 1, Table S1). Primer sequences spanning exon-exon junctions were either adopted from published literature or newly developed using NCBI’s Primer-BLAST platform. All designed primers were < 30 nt in length and generated amplification products <150 bp. The primer validation process was followed by Akram et al. (2024). Briefly, primer validation was performed using a QuantStudio 6 Real-Time PCR instrument (Thermo Fisher Scientific), where primer efficiency and specificity were evaluated through three-fold serial dilutions of a composite cDNA sample representing all experimental groups. Post-amplification verification included both agarose gel electrophoresis (confirming single-band products) and melting curve analysis during qPCR.

### Reverse transcription and preamplification

cDNA synthesis was performed on 50 ng total RNA using a commercial reverse transcription master mix (Standard BioTools, South San Francisco, CA) according to the manufacturer’s specifications. For target preamplification, a pooled primer solution was prepared by combining equal amounts (100 µM, 1 µL each) of forward and reverse primers, adjusting to 400 µL with Tris-EDTA buffer (Thermo Fisher Scientific). This primer pool was then added to Fluidigm PreAmp Mastermix (Standard BioTools) to create the preamplification reaction mixture. In a 96-well thermal cycler, 3.75 µL of the preamplification mixture was combined with 1.25 µL of cDNA template. The amplification protocol consisted of an initial denaturation at 95 °C for 2 min, followed by 14 cycles of denaturation at 95 °C for 15 seconds and annealing/extension at 60 °C for 4 min. To ensure reaction specificity, residual primers were enzymatically degraded using Exonuclease I (New England Biolabs, MA). The final products underwent ten-fold dilution in Tris-EDTA buffer and were preserved at −20 °C for subsequent analysis.

### High-throughput qPCR

Quantitative PCR analysis was performed using the BioMark HD system (Standard BioTools) with 96.96 Dynamic Array integrated fluidic circuits (**IFC**) following established protocols (Akram et al., 2024). A normalized cDNA pool was created by combining equal aliquots (10 µL) from all individual samples, which were subsequently pre-amplified and serially diluted three-fold to generate standard curves for primer efficiency determination on IFC. All reactions included non-template controls to monitor potential contamination. The sample mixture consisted of 0.25 µL 20 × DNA Binding Dye (Standard BioTools) and 2.5 µL 2 × SSoFast EvaGreen Supermix with low ROX (Bio-Rad), while the assay mixture contained 2.5 μL 2 × Assay Loading Reagent (Standard BioTools) and 2.25 μL 1 × TE buffer (TEKnova, Hollister, CA). Amplification was carried out under the following conditions: initial denaturation at 95 °C for 60 s, followed by 30 cycles of denaturation at 96 °C for 5 s and combined annealing/extension at 60 °C for 20 s. Fluorescence data were collected using SBI Real-Time PCR software (v1.0.2, Standard BioTools), with relative quantification performed against standard curves generated. Reference gene stability was evaluated using NormFinder (Andersen et al., 2004), identifying two optimal housekeeping genes (YWHAZ, B-ACTIN) that demonstrated consistent expression. Final relative gene expression values were calculated using the Pfaffl method (Pfaffl, 2001) and normalized to the geometric mean of these reference genes, ensuring accurate quantification of target transcript levels.

### Serum tryptophan metabolites

Serum aliquots (250 μL) from D 7 and D 14 were each combined with 100 μL of 60% perchloric acid and 100 μL of an internal standard solution of 3-nitrotyrosine (60 ng/μL). The mixture was brought to a 750 μL final volume using ultrapure water in 1.5 mL microcentrifuge tubes. Following immediate vortex mixing for 10 s and 5-minute ice incubation, samples underwent centrifugation at 14000 × g for 10 min at 4 °C. The cleared supernatant was membrane-filtered using 0.22 μm nylon filters into autosampler vials for 4 °C storage prior to analysis.

The analysis was performed using a Shimadzu UHPLC system with dual detection via photodiode array (PDA, SPD-M40) and native fluorescence. Separation was achieved on a Shim-pack XR-ODS analytical column (100 mm × 3.0 mm, 2.2 μm) maintained at 40 °C, protected by a guard column system (Shim-pack GVP-ODS, 10 mm × 4.6 mm, 5 μm). The mobile phase consisted of 10 mM sodium acetate buffer (pH 4.8) and acetonitrile, delivered at 0.7 mL/min with gradient elution. For detection, the PDA monitored the 190-800 nm range with primary detection at 360 nm (8 nm slit width, 40 °C cell temperature). Fluorescence detection (RF-20Axs) employed 280 nm excitation and 370 nm emission (30 °C cell temperature, × 4 gain, medium sensitivity, 1 s response time). Sample injection volumes were 30 μL.

All reference standards (tryptophan, kynurenic acid, and indoleacetic acid) were obtained from Sigma-Aldrich. Stock solutions were prepared in appropriate solvents: DMSO (kynurenic acid, 3.9 mg/mL), sodium phosphate buffer (tryptophan, 4.5 mg/mL), ethanol (indoleacetic acid, 4 mg/mL). The prepared stock solutions were stored at −20 °C until use. For quantitative analysis, calibration curves were established for each analyte by correlating peak areas with known concentrations, utilizing retention times and mass spectral data for compound identification.

### Microbial data acquisition

The cecal microbiota data used in this study were obtained from our previous research (Zhao et al., 2025a) and originated from the same experimental cohort. Briefly, 16S rRNA gene sequencing was performed on cecal samples collected from broiler chickens on D 7 and D 14 following fecal microbiota transplantation. The sequencing data are publicly available under the NCBI Bioproject accession number PRJNA1299803. For the current analysis, we specifically focused on the dominant bacterial genera, defined as those with a mean relative abundance > 1% across all samples. This criterion resulted in the selection of 18 genera, which served as the independent variables for the subsequent Partial Least Squares Regression (**PLSR**) modeling.

### Statistical analysis

Body weight, ileal histomorphology, gene expression, and tryptophan metabolite data were analyzed using R Studio (v4.2.3, R Foundation, Vienna, Austria). Data normality was confirmed via the Shapiro-Wilk test prior to analysis. Group differences were assessed using a one-way ANOVA followed by Tukey’s post-hoc test, with statistical significance defined as *P* < 0.05. For gene expression data, *P*-values were adjusted for multiple comparisons using the Benjamini-Hochberg false discovery rate method, where significance was defined as an adjusted *P* < 0.05.

To elucidate the multivariate relationships between the gut microbiota and host parameters, including ileal histomorphology, ileal gene expression, and serum tryptophan, PLSR models were constructed in R using the mixOmics package. The predictor matrix (X) consisted 18 dominant bacterial genera (mean relative abundance > 1%) (Zhao et al., 2025a), while the response matrix (Y) was composed of indices that exhibited significant differences among groups in the present study. To identify the most influential microbial features driving the variation in host parameters, Variable Importance in Projection (**VIP**) scores were calculated from the PLSR model, and genera with a VIP > 1.0 were considered key contributors.

## Results

### Body weight

**Fig. 1:**
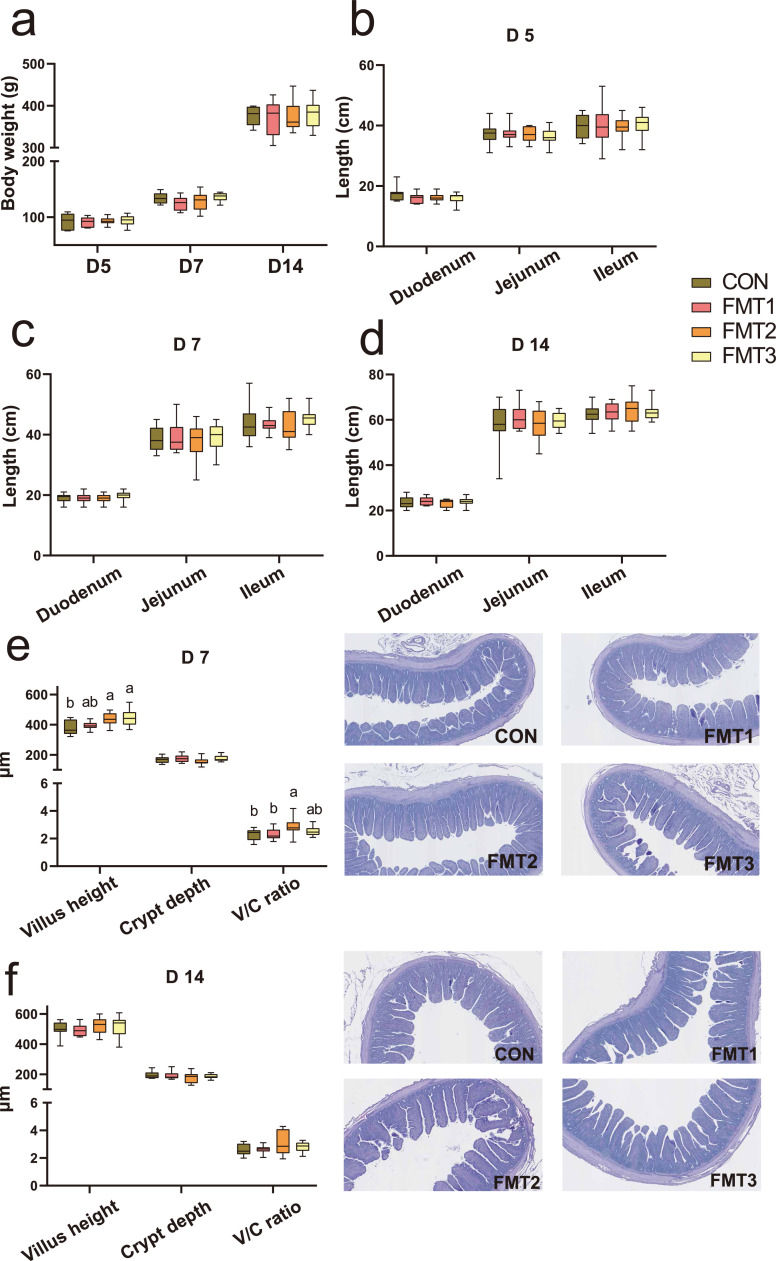
Effects of fecal microbiota transplantation on body weight and intestinal histomorphology of broilers. **(a)** Body weight (g) measured on D 5, D 7, D14. **(b-d)** The length of duodenum, jejunum, and ileum. **(e-f)** Ileal histomorphology, including villus height (cm), crypt depth (cm), and villus height to crypt depth (V/C) ratio. Groups FMT1, FMT2, and FMT3 received inocula from broilers, laying hens, and broiler breeders, respectively. Data are presented as box plots showing the mean ± SEM, n = 10 biological replicates. Significant differences were determined by one-way ANOVA followed by Tukey’s post hoc test, with different lowercase letters indicating statistical significance (*P* < 0.05).

As shown in Fig. 1a, there was no difference between the FMT groups and CON in body weight across all time points (*P* > 0.05).

### Intestinal length and ileal histology

There was no difference in duodenal, jejunal, or ileal length between the FMT groups and the CON group across all time points (Fig. 1b-d; *P* > 0.05). On D 7 (Fig. 1e), the VH in the FMT2 and FMT3 groups was significantly higher than in the CON group (*P* < 0.05), whereas there was no difference between the CON and FMT1 groups (*P* > 0.05). The villus-to-crypt ratio in the FMT2 group was significantly higher than in the CON group (*P* < 0.05), whereas there was no difference between the CON and other FMT groups (*P* > 0.05). On D 14 (Fig. 1f), there was no difference between the CON and FMT groups (*P* > 0.05).

### Ileal gene expression

**Fig. 2:**
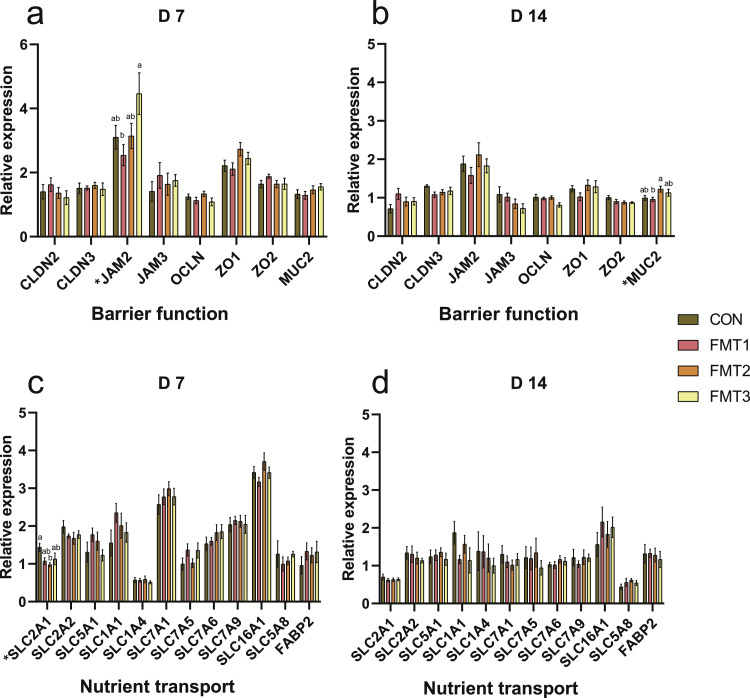
Effects of fecal microbiota transplantation on the expression of genes related to barrier function and nutrient transport in the ileum of broilers. **(a-b)** Relative mRNA expression of barrier function genes measured on D 7 and D 14. **(c-d)** Relative mRNA expression of nutrient transport genes measured on D 7 and D 14. Groups FMT1, FMT2, and FMT3 received inocula from broilers, laying hens, and broiler breeders, respectively. Data are presented as bar charts showing the mean ± SEM, n = 10 biological replicates. Significant differences were determined by one-way ANOVA followed by Tukey’s post hoc test. *P*-values were adjusted using the False Discovery Rate correction (Benjamini-Hochberg). Different lowercase letters indicate statistical significance based on the adjusted *P*-values (*P* < 0.05). *CLDN2*: Claudin 2; *CLDN3*: Claudin 3; *JAM2*: Junctional adhesion molecule 2; *JAM3*: Junctional adhesion molecule 3; *OCLN*: Occludin; *ZO1*: Zonula occludens 1; *ZO2*: Zonula occludens 2; *MUC2*: Mucin 2; *SLC2A1*: Glucose transporter 1; *SLC2A2*: Glucose transporter 2; *SLC5A1*: Sodium/glucose cotransporter 1; *SLC1A1*: Excitatory amino acid carrier 1; *SLC1A4*: Neutral amino acid transporter; *SLC7A1*: Cationic amino acid transporter 1; *SLC7A5*: l-type amino acid transporter 1; *SLC7A6*: y + L amino acid transporter 2; *SLC7A9*: b(0,+) amino acid transporter; *SLC16A1*: Monocarboxylate transporter 1; *SLC5A8*: Sodium-coupled monocarboxylate transporter 1; *FABP2*: Fatty acid binding protein 2.

***Barrier Function.*** On D 7 (Fig. 2a), the relative mRNA expression levels of all evaluated barrier function genes showed no significant differences between the CON group and any of the individual FMT groups (*P* > 0.05). However, among the FMT treatments, the expression of junctional adhesion molecule 2 (***JAM2***) in the FMT3 group was significantly higher than that in the FMT1 group (*P* < 0.05). Similarly, on D 14 (Fig. 2b), no significant differences were observed in the expression of all tested barrier function genes between the CON group and the FMT groups (*P* > 0.05). However, within the FMT treatments, the expression of mucin 2 (***MUC2***) in the FMT2 group was significantly higher compared to the FMT1 group (*P* < 0.05).

***Nutrient Transport*.** On D 7 (Fig. 2c), the expression of glucose transporter 1 (***SLC2A1***) was significantly lower in the FMT2 group compared to the CON group (*P* < 0.05). On D 14 (Fig. 2d), there was no difference between the FMT groups and the CON group on the nutrient transport related gene expression (*P* > 0.05).

**Fig. 3:**
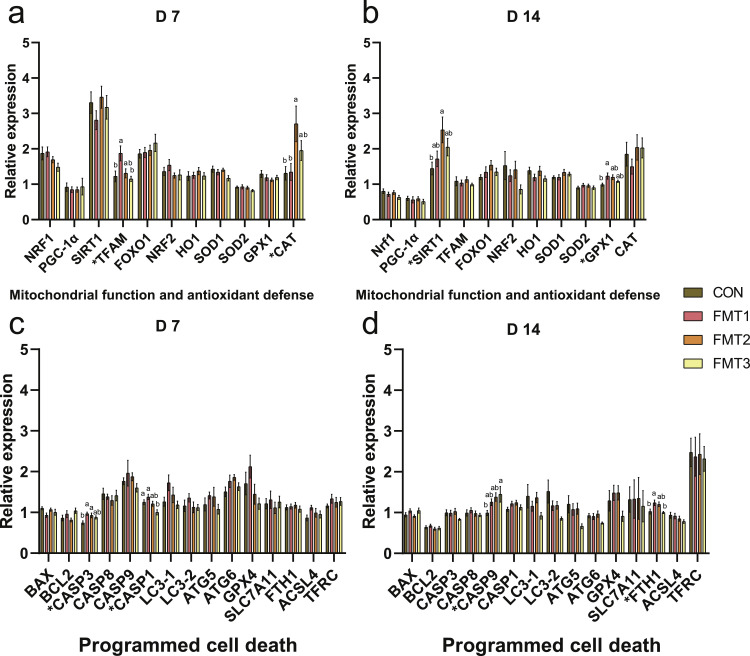
Effects of fecal microbiota transplantation on the expression of genes related to mitochondrial function, antioxidant defense, and programmed cell death in the ileum of broilers. **(a-b)** Relative mRNA expression of mitochondrial function and antioxidant defense genes measured on D 7 and D 14. **(c-d)** Relative mRNA expression of programmed cell death genes measured on D 7 and D 14. Groups FMT1, FMT2, and FMT3 received inocula from broilers, laying hens, and broiler breeders, respectively. Data are presented as bar charts showing the mean ± SEM, n = 10 biological replicates. Significant differences were determined by one-way ANOVA followed by Tukey’s post hoc test. *P*-values were adjusted using the False Discovery Rate correction (Benjamini-Hochberg). Different lowercase letters indicate statistical significance based on the adjusted *P*-values (*P* < 0.05). *NRF1*: Nuclear respiratory factor 1; *PGC-1α*: Peroxisome proliferator-activated receptor gamma coactivator 1-alpha; *SIRT1*: Sirtuin 1; *TFAM*: Mitochondrial transcription factor A; *FOXO1*: Forkhead box O1; *NRF2*: Nuclear factor erythroid 2-related factor 2; *HO1*: Heme oxygenase 1; *SOD1*: Superoxide dismutase 1; *SOD2*: Superoxide dismutase 2; *GPX1*: Glutathione peroxidase 1; *CAT*: Catalase; *BAX*: Bcl-2-associated X protein; *BCL2*: B-cell lymphoma 2; *CASP1*: Caspase 1; *CASP3*: Caspase 3; *CASP8*: Caspase 8; *CASP9*: Caspase 9; *LC3*: Microtubule-associated protein 1 light chain 3; *ATG5*: Autophagy related 5; *ATG6*: Autophagy related 6; *GPX4*: Glutathione peroxidase 4; *SLC7A11*: Cystine/glutamate antiporter; *FTH1*: Ferritin heavy chain 1; *ACSL4*: Acyl-CoA synthetase long chain family member 4; *TFRC*: Transferrin receptor.

***Mitochondrial Function and Antioxidant Defense.*** On D 7 (Fig. 3a), the expression of mitochondrial transcription factor A (***TFAM***) in the FMT1 group was significantly higher than in the CON group (*P* < 0.05). The expression of catalase (***CAT****)* was significantly higher in the FMT2 group compared to the CON group (*P* < 0.05). On D 14 (Fig. 3b), the expression of sirtuin 1 (***SIRT1***) in the FMT2 group was significantly higher than in the CON group (*P* < 0.05). The expression of glutathione peroxidase 1 (***GPX1***) in the *FMT1* group was significantly higher than in the CON group (*P* < 0.05).

***Programmed Cell Death.*** On D 7 (Fig. 3c), the expression of caspase 3 (***CASP3***) in the FMT1 and FMT2 groups was significantly higher than in the CON group (*P* < 0.05). The expression of *CASP1* in the FMT3 group was significantly lower than in the CON group (*P* < 0.05). On D 14 (Fig. 3d), the expression of *CASP9* in the FMT3 group was significantly higher than in the CON group (*P* < 0.05). The expression of Ferritin heavy chain 1 (***FTH1***) in the FMT1 group was significantly higher than in the CON group (*P* < 0.05).

**Fig. 4:**
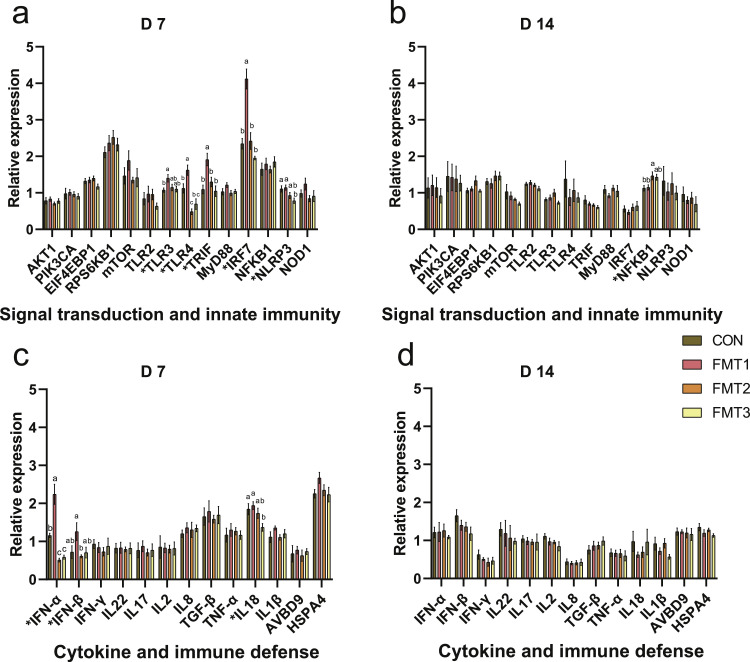
Effects of fecal microbiota transplantation on the expression of genes related to signal transduction, innate immunity, and immune defense in the ileum of broilers. **(a-b)** Relative mRNA expression of signal transduction and innate immunity genes measured on D 7 and D 14. **(c-d)** Relative mRNA expression of cytokine and immune defense genes measured on D 7 and D 14. Groups FMT1, FMT2, and FMT3 received inocula from broilers, laying hens, and broiler breeders, respectively. Data are presented as bar charts showing the mean ± SEM, n = 10 biological replicates. Significant differences were determined by one-way ANOVA followed by Tukey’s post hoc test. *P*-values were adjusted using the False Discovery Rate correction (Benjamini-Hochberg). Different lowercase letters indicate statistical significance based on the adjusted *P*-values (*P* < 0.05). *AKT1*: AKT serine/threonine kinase 1; *PIK3CA*: Phosphatidylinositol-4,5-bisphosphate 3-kinase catalytic subunit alpha; *EIF4EBP1*: Eukaryotic translation initiation factor 4E binding protein 1; *RPS6KB1*: Ribosomal protein S6 kinase B1; *mTOR*: Mechanistic target of rapamycin kinase; *TLR2*: Toll-like receptor 2; *TLR3*: Toll-like receptor 3; *TLR4*: Toll-like receptor 4; *TRIF*: TIR-domain-containing adapter-inducing interferon-β; *MyD88*: Myeloid differentiation primary response 88; *IRF7*: Interferon regulatory factor 7; *NFKB1*: Nuclear factor kappa B subunit 1; *NLRP3*: NLR family pyrin domain containing 3; *NOD1*: Nucleotide binding oligomerization domain containing 1; *IFNα*: Interferon alpha; *IFNβ*: Interferon beta; *IFNγ*: Interferon gamma; *IL22*: Interleukin 22; *IL17*: Interleukin 17; *IL2*: Interleukin 2; *IL8*: Interleukin 8; *TGFβ*: Transforming growth factor beta; *TNFα*: Tumor necrosis factor alpha; *IL18*: Interleukin 18; *IL1β*: Interleukin 1 beta; *AVBD9*: Avian beta-defensin 9; *HSPA4*: Heat shock protein family A member 4.

***Signal Transduction and Innate Immunity.*** On D 7 (Fig. 4a), the expression of toll-like receptor 3 (***TLR3***) in the FMT1 group was significantly higher than in the CON group (*P* < 0.05). The expression of *TLR4* was significantly higher in the FMT1 group, whereas it was significantly lower in the FMT2 group compared to the CON group (*P* < 0.05). The expression of interferon regulatory factor 7 (***IRF7***) and TIR-domain-containing adapter-inducing interferon-β (***TRIF***) was significantly higher in the FMT1 group compared to the CON group (*P* < 0.05). The expression of NLR family pyrin domain containing 3 (***NLRP3***) in the FMT3 group was significantly lower than in the CON group (*P* < 0.05). On D 14 (Fig. 4b), the expression of nuclear factor kappa B subunit 1 (***NFKB1***) in the FMT2 group was significantly higher than in the CON group (*P* < 0.05).

***Cytokine and Immune Defense.*** On D 7 (Fig. 4c), the expression of interferon alpha (***IFN-α***) was significantly higher in the FMT1 group but lower in the FMT2 and FMT3 groups compared to the CON group (*P* < 0.05). The expression of interleukin 18 (***IL-18***) in the FMT3 group was significantly lower compared to the CON group (*P* < 0.05). On D 14 (Fig. 4d), there was no difference in the expression of cytokine-related genes between the CON and FMT groups (*P* > 0.05).

**Fig. 5:**
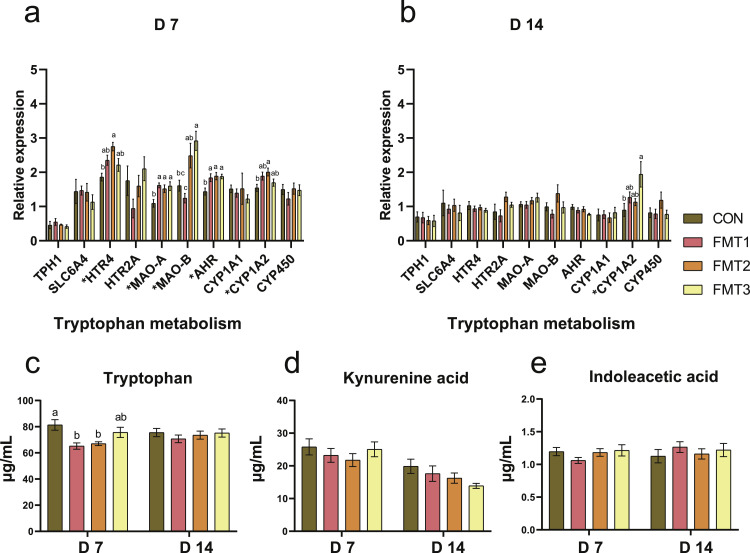
Effects of fecal microbiota transplantation on the expression of genes related to tryptophan metabolism in the ileum and serum tryptophan metabolite concentrations of broilers. **(a-b)** Relative mRNA expression of tryptophan metabolism genes measured on D 7 and D 14. **(c-e)** Serum concentrations (µg/mL) of tryptophan related metabolites measured on D 7 and D 14. Groups FMT1, FMT2, and FMT3 received inocula from broilers, laying hens, and broiler breeders, respectively. Data are presented as bar charts showing the mean ± SEM, n = 10 biological replicates. Significant differences were determined by one-way ANOVA followed by Tukey’s post hoc test. *P*-values were adjusted using the False Discovery Rate correction (Benjamini-Hochberg). Different lowercase letters indicate statistical significance based on the adjusted *P*-values (*P* < 0.05). *TPH1*: Tryptophan hydroxylase 1; *SLC6A4*: Serotonin transporter; *HTR4*: 5-Hydroxytryptamine receptor 4; *HTR2A*: 5-Hydroxytryptamine receptor 2A; *MAO-A*: Monoamine oxidase A; *MAO-B*: Monoamine oxidase B; *AHR*: Aryl hydrocarbon receptor; *CYP1A1*: Cytochrome P450 family 1 subfamily A member 1; *CYP1A2*: Cytochrome P450 family 1 subfamily A member 2; *CYP450*: Cytochrome P450.

***Tryptophan Metabolism*.** On D 7 (Fig. 5a), the expression of 5-hydroxytryptamine receptor 4 (***HTR4***) was significantly higher in the FMT2 group compared to the CON group (*P* < 0.05). The expression of monoamine oxidase A (***MAO-A***) and aryl hydrocarbon receptor (***AHR***) in all FMT groups was significantly higher than in the CON group (*P* < 0.05). The expression of *MAO-B* in the FMT3 group was significantly higher than in the CON group (*P* < 0.05). The expression of cytochrome P450 family 1 subfamily A member 2 (***CYP1A2***) in the FMT2 group was significantly higher than in the CON group (*P* < 0.05). On D 14 (Fig. 5b), the expression of *CYP1A2* in the FMT3 group was significantly higher than in the CON group (*P* < 0.05).

### Serum Tryptophan Metabolites

On D 7, serum tryptophan concentration (Fig. 5c) in FMT1 and FMT2 was significantly lower than in CON (*P* < 0.05). No significant differences were observed between FMT groups and CON in serum kynurenic acid or indoleacetic acid (Fig. 5d and e; *P* > 0.05).

On D 14, no significant differences were found between FMT and CON in serum tryptophan, kynurenic acid, or indoleacetic acid (Fig. 5c-e; *P* > 0.05).

### Integrative analysis of the gut microbiota and host parameters

**Fig. 6:**
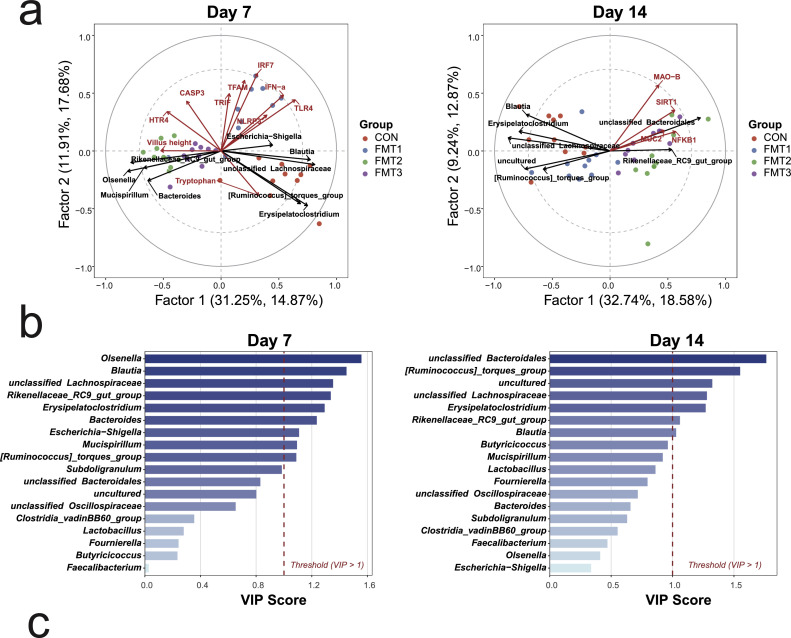
Multivariate associations between dominant cecal genera and host physiological parameters in broilers. **(a)** Partial Least Squares Regression (**PLSR**) biplots visualizing the covariance between dominant cecal genera and host parameters. **(b)** Variable Importance in Projection (**VIP**) scores derived from the PLSR model to identify genera playing a pivotal role in driving host variations. Groups FMT1, FMT2, and FMT3 received inocula from broilers, laying hens, and broiler breeders, respectively. PLSR models were constructed using 18 dominant bacterial genera (X matrix) and significantly changed host indices (Y matrix). VIP scores derived from the PLSR model to identify genera in driving host variations, a threshold of VIP > 1.0 indicates influential contributors.

The PLSR model was employed to investigate the potential interactions between the 18 dominant bacterial genera (mean relative abundance > 1%) and host physiological parameters. On D 7 (Fig. 6a), the PLSR model indicated that these dominant genera explained 32.55% (R^2^Y) of the variance in the host parameters. The PLSR biplot revealed that the genera *Escherichia-Shigella, Blautia*, and unclassified *Lachnospiraceae* were projected in the same direction as key pro-inflammatory markers, including *TLR4* and *IFN-α*. In contrast, *Olsenella, Mucispirillum, Bacteroides*, and *Rikenellaceae_RC9_gut_group* were situated in the opposite quadrant to these inflammatory markers, but were oriented in the same direction as *HTR4* and VH. Besides, *[Ruminococcus]_torques_group* and *Erysipelatoclostridium* were projected in the same direction as serum tryptophan. Nine key bacterial genera were identified through VIP scores (Fig. 6b; VIP > 1.0), among which *Olsenella, Blautia*, and unclassified *Lachnospiraceae* were the microbial members that contributed most significantly to the differences in host gene expression.

On D 14 (Fig. 6a), the PLSR model indicated that the dominant genera explained 31.45% (R^2^Y) of the variance in the host parameters. The PLSR biplot showed that unclassified *Bacteroidales* and *Rikenellaceae_RC9_gut_group* were oriented in the same direction as *MAO-B, SIRT1, MUC2*, and *NFKB1*, whereas *Blautia, Erysipelatoclostridium*, unclassified *Lachnospiraceae*, and *[Ruminococcus]_torques_group* were projected in the opposite direction of these genes. Seven key bacterial genera were screened through VIP scores (Fig. 6b), with unclassified *Bacteroidales* and *[Ruminococcus]_torques_group* being the microbial members that contributed most to the differences in host gene expression.

## Discussion

The gut microbiota of chickens is largely independent of host genetic factors but is significantly influenced by environmental and management factors, leading to considerable variability (Dai et al., 2022; Wen et al., 2019). FMT is a promising intervention for reshaping gut microbial composition (Zhao et al., 2025b). Given that early microbial colonization plays a pivotal role in long-term gut health (Dai et al., 2022; Gensollen et al., 2016), this study aimed to determine whether early microbial modulation could influence broiler growth performance and intestinal health, and more importantly, whether such effects are driven by differences in donor microbial composition.

In contrast to studies reporting FMT-induced weight gain (Chen et al., 2024; Elokil et al., 2022; Fu et al., 2022; Khalid et al., 2024; Ma et al., 2023; Siegerstetter et al., 2018; Yu et al., 2021; Zhang et al., 2022b) or reduction (Chen et al., 2025a), our results demonstrated no significant effect on broiler body weight, consistent with findings reported in other studies (Yan et al., 2021; Zhang et al., 2023). The effects of FMT on broiler body weight remain inconsistent across studies, which may be attributed to variations in donor microbiota composition and the resilience of the recipient’s pre-existing microbiota (Lee et al., 2019; Rychlik, 2020; Zhao et al., 2025b). For example, recipients receiving microbiota from feed-efficient chickens showed increased body weight (Siegerstetter et al., 2018). However, transplantation of microbiota from aged chickens improved fearfulness without affecting growth performance (Yan et al., 2021). Furthermore, weight-associated core microbiota, such as *Lactobacillus, Bifidobacterium*, and *Lactococcus*, may fail to establish functional niches in newly hatched chicks due to unfavorable ecological competition, thereby limiting their phenotypic expression (Han et al., 2016; Zhang et al., 2023; Zhao et al., 2025b).

In the present study, no changes in intestinal length were observed during the first two weeks. This finding contrasts with previous studies that FMT increased jejunal length in broilers (Liu et al., 2025; Ma et al., 2023). The underlying mechanism by which FMT may influence intestinal growth is potentially mediated by beneficial microbial metabolites, such as SCFA, cofactors, and vitamins, which can indirectly promote intestinal development (Zhao et al., 2025b). The absence of this effect in this study may be attributed to the limited 2-week rearing period, which was likely insufficient for cumulative microbial colonization and metabolite production to reach thresholds capable of inducing detectable intestinal length changes (Zhang et al., 2023). Moreover, the rapid intestinal growth during the first 14 days post-hatch may have masked subtle FMT-induced effects, thereby limiting observable differences in length (Dai et al., 2022). Extending the gavage period and prolonging the observation window to a standard 35-42 day broiler production cycle may be necessary to reveal more profound and lasting physiological and microbial shifts. On the contrary, by D 7, the FMT2 group exhibited significantly higher VH and V/C compared to the control group. Notably, on D 7, the PLSR model identified *Olsenella, Mucispirillum, Bacteroides*, and the *Rikenellaceae_RC9_gut_group* as the most important discriminatory features, which were positively associated with VH. These findings demonstrate that FMT had the potential to promote intestinal epithelial development and enhanced absorption capacity in broilers. This effect may be driven by the introduction of specific beneficial microbes that modulate gut microbiota composition, potentially leading to increased production of SCFA, which are known to stimulate epithelial cell proliferation and increase VH (Zhao et al., 2025b; Zhou et al., 2025). However, these outcomes appear to vary depending on the donor’s microbial composition. Similarly, Zhang et al. (2022a) observed that FMT improved villus structure, which was likely linked to the abundance of *Lactobacillus*. In contrast, other studies indicate that FMT leading to stress in the recipient may result in reduced VH (Chen et al., 2025a, 2025b). In this study, FMT had no effect on ileal structure in recipient broilers on D 14, indicating that FMT’s impact is possibly time-dependent, and single or short-term FMT may be insufficient for sustained morphological improvements.

In our study, FMT did not significantly alter ileal *JAM2* expression on D 7 compared with the control group, although expression in the FMT3 group was higher than in FMT1. It has been reported that *JAM2* expression can be upregulated by glyceraldehyde-3-phosphate dehydrogenase secreted by *Lactobacillus johnsonii* (Bai et al., 2022). Although our data indicated a higher relative abundance of the *Lactobacillus* in the FMT3 group (Zhao et al., 2025a), the specific association between *JAM2* and microbial species requires further verification at the species level. Contrary to previous reports that FMT enhanced barrier function via mucin upregulation (Song et al., 2024; Zaytsoff et al., 2022), we observed no significant improvement compared to the control. However, FMT2 induced higher *MUC2* expression than FMT1 on D 14. Given MUC2’s role in forming the protective mucus layer against pathogens (Zhao et al., 2025b), our results point to donor-dependent impact on barrier function. Intestinal barrier function directly affects nutrient absorption because tight junctions and mucus layers regulate the passage of nutrients across the epithelium (Chelakkot et al., 2018). Our study found that FMT2 significantly reduced *SLC2A1* expression in the ileum on D 7, suggesting a potential decrease in glucose transport from enterocytes to the bloodstream (Portincasa et al., 2022). This finding aligns with our previous observation that the FMT2 microbiota exhibited significantly enriched carbohydrate biosynthesis pathways (Zhao et al., 2025a). This suggests that the intervention shifted microbial metabolism to produce SCFAs, which serve as alternative energy substrates for epithelial cells, thereby reducing the host’s reliance on SLC2A1 transport (Portincasa et al., 2022). Interestingly, the reduced *SLC2A1* expression contrasts with the significantly higher VH and V/C ratio in the FMT2 group on D 7. This indicates that FMT2 also optimizes glucose and nutrient absorption efficiency through improved intestinal histomorphology, reducing dependence on high glucose transporter expression (Chelakkot et al., 2018; Portincasa et al., 2022).

FMT has been recognized for its numerous beneficial effects on chicken intestinal health, though some studies suggest it may induce intestinal stress (Chen et al., 2025a, 2025b). Given this duality, and because mitochondrial function and antioxidant capacity directly influence energy metabolism and oxidative stress in intestinal epithelial cells (Haque et al., 2024), we examined markers of these pathways. On D 7, *TFAM* was significantly upregulated in FMT1. TFAM is a key regulator of mitochondrial DNA replication and transcription, and likely reflects enhanced mitochondrial biogenesis and improved epithelial energy metabolism in FMT1 (Kong et al., 2022). Similarly, Chen et al. (2024) reported that FMT affects the expression of mitochondrial biogenesis-related genes in cardiomyocytes. Since microbial metabolites regulate the mitochondrial respiratory chain and ATP production (Saint-Georges-Chaumet and Edeas, 2016), these findings indicate that FMT can transfer microbiota-driven effects on mitochondrial function to the host. We speculate that the distinct effects observed in FMT1 may be associated with its unique donor microbial composition. Although the specific microbial metabolites responsible for this cross-talk were not characterized in the current study, our analysis supports a strong microbiota-host association. Moreover, the PLSR biplot revealed that the FMT1 microbiota aligned with the *TFAM* vector, while the microbiota of FMT2 and FMT3 clustered in the opposite direction. These findings indicate that donor microbiota composition differentially modulates recipient mitochondrial function. Likewise, the upregulation of *CAT* in the FMT2 group on D 7 indicates enhanced antioxidant defense, with CAT mitigating oxidative stress by decomposing hydrogen peroxide, thus protecting intestinal epithelial cells from oxidative damage (Kunst et al., 2023). Similarly, Zhou et al. (2025) reported that FMT increased serum CAT levels, which may be related to the core commensal *Lactobacillus gallinarum*. The significant upregulation of *SIRT1* expression in the FMT2 group and *GPX1* in the FMT1 group on D 14 further supports FMT’s influence on intestinal antioxidant capacity. The importance of these markers lies in their respective roles in maintaining redox homeostasis. Specifically, SIRT1 regulates mitochondrial biogenesis and antioxidant defense gene expression through deacetylation, enhancing cellular resistance to oxidative stress (Kong et al., 2022; Kunst et al., 2023). In contrast, GPX1 acts as an important antioxidant enzyme that directly scavenges reactive oxygen species to mitigate oxidative damage (Kunst et al., 2023). Notably, the PLSR model indicated that the increased expression of *SIRT1* was positively correlated with the relative abundances of *Rikenellaceae_RC9_gut_group* and unclassified *Bacteroidales*. This finding aligns with prior reports (Jin et al., 2024) and suggest that these specific genera may serve as key drivers of the elevated *SIRT1* expression observed in the FMT2 group.

In this study, FMT1 was associated with significantly increased gene expression of *TLR3, TLR4, TRIF*, and *IRF7*, together with higher *INF*-*α* expression on D 7 compared to CON. These genes are well-known components of pattern recognition and interferon signaling pathways, where TLR3, TLR4, and TRIF function as key receptors and adaptors for recognizing viral double-stranded RNA and bacterial lipopolysaccharide (**LPS**), respectively (Kawai and Akira, 2011). Their activation leads to IRF7-mediated induction of interferons, enhancing antiviral and antibacterial responses (Kawai and Akira, 2011). The observed expression profile suggests that FMT1 may have triggered robust immune activation. This modulation could be driven by the enrichment of specific bacterial genera, including *Escherichia-Shigella, Blautia*, and unclassified *Lachnospiraceae*, which have been closely linked to the immune system in broilers (Liu et al., 2023), and are known to contain immunostimulatory members capable of enhancing host defenses (Kawai and Akira, 2011; Yin et al., 2023). Similarly, Chen et al. (2025b) also found intestinal inflammation after FMT, accompanied by increased intestinal *TLR4* gene expression. This heightened immune response may challenge the colonization of exogenous microbes from FMT. For instance, elevated *TLR4* expression likely triggers local inflammation by recognizing LPS in FMT-derived microbiota, potentially limiting the survival or function of sensitive microbial populations (Yin et al., 2023). Conversely, the other two FMT groups showed downregulated expression of signaling and inflammation-related genes on D 7. As indicated by the PLSR model, the microbial clusters in FMT2 and FMT3 were inversely correlated with these gene expression patterns, suggesting a low immune-stress environment that may facilitate the colonization of exogenous microbiota (Zhao et al., 2025b). These conditions likely create a favorable niche for the proliferation and metabolic activity of potentially beneficial genera, such as *Olsenella, Mucispirillum, Bacteroides*, and *Rikenellaceae_RC9_gut_group*. However, by D 14, only the FMT2 group showed upregulated *NFKB1* gene expression, with no significant changes in other signaling or inflammation-related genes, indicating that immune stress effects on FMT outcomes may have diminished over time. This could be associated with gut microbiota maturation, contributing to a balanced intestinal microecology (Dai et al., 2022; Zhao et al., 2025a, 2025b).

The differential regulation of programmed cell death related genes highlights distinct mechanisms among the recipient groups. In the FMT1 group, the upregulation of ileal *CASP3* expression on D 7 appears to be driven by immune stress (Kuo et al., 2019). Given that pattern recognition receptors (*TLR3, TLR4*) were activated in FMT1, the observed elevation in *CASP3* likely stems from enhanced innate immune signaling via TLR/NF-κB pathways, suggesting that it drives apoptosis as a response to intestinal inflammation (Kawai and Akira, 2011; Kuo et al., 2019). This state of sustained stress is further evidenced by the significant upregulation of *FTH1* on D 14. While *FTH1* typically protects against oxidative damage by storing iron, its excessive and prolonged upregulation in FMT1 likely reflects a persistent response to the oxidative stress initially observed on D 7, potentially disrupting iron metabolism and cellular function (Xu et al., 2021). Conversely, the FMT2 group displayed a distinct profile where significantly upregulated *CASP3* expression on D 7 aligned with increased VH and V/C ratios. This alignment suggests that, rather than signaling damage, FMT2 promoted intestinal epithelial cell turnover (Vriz et al., 2014). In this context, apoptosis serves as a regulated mechanism to remove aging cells and accommodate the rapid proliferation of villi, contributing to the optimized histomorphology observed. Finally, the FMT3 group exhibited a protective mechanism characterized by the suppression of inflammatory cell death and immune stress. Specifically, FMT3 significantly downregulated *CASP1, NLRP3*, and *IL18*, indicating the inhibition of NLRP3 inflammasome-mediated pyroptosis (Miao et al., 2011). This mitigation of pro-inflammatory death allows for the maintenance of barrier integrity (Miao et al., 2011), as reflected by elevated *JAM2* expression. Although *CASP9* was upregulated on D 14 in FMT3, this potentially represents a non-inflammatory mitochondrial pathway to clear damaged cells and maintain barrier homeostasis (Chelakkot et al., 2018; Miao et al., 2011), distinct from the immune-driven apoptosis seen in FMT1. However, the mechanistic insights presented in this study rely on mRNA expression profiles. Because transcript levels do not necessarily reflect actual protein abundance or functional activity, future investigations must incorporate targeted protein-level validation to further verify the impact of FMT on intestinal health.

Tryptophan is an essential amino acid in broilers that can be metabolized via the kynurenine, serotonin (**5-HT**), and indole pathway, which are critical for broiler gut health and growth performance (Agus et al., 2018). In the FMT2 group, the significantly reduced serum tryptophan levels were accompanied by the upregulation of ileal *CYP1A2* and *AHR*. Given that CYP1A2 is a downstream target of AHR, its upregulation suggests activation of AHR signaling, potentially driven by tryptophan-derived microbial metabolites. This may reflect enhanced tryptophan metabolic turnover, which likely contributes to the decrease in serum tryptophan levels. This metabolic shift may contribute to intestinal immune homeostasis and barrier integrity (Agus et al., 2018; Gao et al., 2020). Notably, PLSR model suggests the reduction in serum tryptophan may be associated with *[Ruminococcus]_torques_group*, which has been previously identified as a negative correlate of tryptophan levels (Huang et al., 2024). In parallel, FMT appeared to enhance 5-HT turnover, as evidenced by the upregulation of *MAO-A* in the FMT1 and FMT2 groups, which contributed to tryptophan consumption (Agus et al., 2018; Zhao et al., 2025b). Since tryptophan is the precursor for 5-HT, its increased breakdown may require greater utilization of tryptophan to replenish the 5-HT pool. This modulation appears linked to *Erysipelatoclostridium* as indicated by the PLSR model, consistent with previous reports that this genus influences 5-HT metabolism (Bloemendaal et al., 2023). Simultaneously, the FMT2 group showed significantly upregulated ileal *HTR4* expression, which is a G protein-coupled receptor that mediates 5-HT-induced stimulation of intestinal motility and secretion (Agus et al., 2018). Similarly, Fu et al. (2022) found that the gut serotonergic systems were activated in the FMT group, accompanied by increased concentrations of secretory immunoglobulin A and IL-10 in the intestine and plasma, which may have induced anti-inflammatory effects in the broiler recipients. Importantly, FMT’s effects on tryptophan metabolism diminished by D 14, further demonstrating FMT has time effects on recipients.

## Conclusion

In conclusion, this study demonstrates that early FMT modulates broiler intestinal health through donor-dependent mechanisms involving immune stress, programmed cell death, antioxidant protection, and tryptophan metabolism. Although FMT did not alter growth performance or intestinal length within the first two weeks, it significantly influenced gut health through specific pathways linked to the donor sources. Specifically, the microbiota from FMT1 (derived from broilers) induced innate immune activation via TLR signaling and oxidative stress and consequently drove apoptosis and mitochondrial biogenesis as adaptive responses. In contrast, FMT2 (derived from laying hens) improved intestinal histomorphology and antioxidant defense. These benefits were linked to shifts in tryptophan metabolism, reflected by changes in AHR signaling and serotonin turnover, as well as a reduced reliance on glucose transporters. Meanwhile, FMT3 (derived from broiler breeders) exhibited a suppression of NLRP3 inflammasome-mediated pyroptosis and enhanced barrier integrity. Importantly, PLSR analysis confirmed these microbiota-host interactions. On D 7, nine key bacterial genera (VIP > 1) were identified, explaining over 30% of the variance in the observed changes. Among them, *Olsenella, Blautia*, and unclassified *Lachnospiraceae* contributed most significantly to the observed host responses. However, these distinct effects were diminished by D 14, indicating the time-dependent influence of early FMT, which requires optimized strategies for sustained benefits. While this study identifies key gene-regulatory networks, future research integrating multi-omics and functional validation is necessary to fully elucidate the cross-talk between donor microbiota and host intestinal health.

## Data availability

All data generated or analyzed during this study are included in this published article and its supplementary information file.

## CRediT authorship contribution statement

**Haoran Zhao:** Writing – review & editing, Writing – original draft, Visualization, Validation, Methodology, Investigation, Funding acquisition, Formal analysis, Data curation, Conceptualization. **Muhammad Zeeshan Akram:** Writing – review & editing, Investigation. **Luke Comer:** Writing – review & editing, Investigation. **Matthias Corion:** Writing – review & editing. **Elena Fako:** Writing – review & editing. **Nadia Everaert:** Writing – review & editing, Funding acquisition, Conceptualization.

## Disclosures

The authors declare the following financial interests/personal relationships which may be considered as potential competing interests:

Haoran Zhao reports financial support was provided by China Scholarship Council. If there are other authors, they declare that they have no known competing financial interests or personal relationships that could have appeared to influence the work reported in this paper.

## Appendix Supplementary materials

Image, application 1

## Footnotes

Supplementary material associated with this article can be found, in the online version, at doi:10.1016/j.psj.2026.106999.
